# P-1158. Surgical Outcomes Following *Paenibacillus* Infant Infectious Hydrocephalus

**DOI:** 10.1093/ofid/ofae631.1344

**Published:** 2025-01-29

**Authors:** Jessica E Ericson, Davis Natukwatsa, Peter Ssenyonga, Justin Onen, John Mugamba, Ronald Mulondo, Sarah Morton, Kelsey Templeton, Christine Hehnly, Edith Mbabazi-Kabachelor, Abhaya Kulkarni, Benjamin Warf, James Broach, Joseph Paulson, Steven Schiff

**Affiliations:** Penn State College of Medicine, Hershey, Pennsylvania; CURE Children's Hospital of Uganda, Mbale, Mbale, Uganda; CURE Children's Hospital of Uganda, Mbale, Mbale, Uganda; CURE Children's Hospital of Uganda, Mbale, Mbale, Uganda; NeuroKids, Fairfax, Virginia; CURE Children's Hospital of Uganda, Mbale, Mbale, Uganda; Harvard Medical School, Boston, Massachusetts; Yale University, New Haven, Connecticut; Harvard Children's Hospital, Boston, Connecticut; CURE Children's Hospital of Uganda, Mbale, Mbale, Uganda; University of Toronto, Toronto, Ontario, Canada; Harvard Medical School, Boston, Massachusetts; Penn State College of Medicine, Hershey, Pennsylvania; Genentech Inc, San Francisco, California; Penn State College of Medicine, Hershey, Pennsylvania

## Abstract

**Background:**

We previously identified *Paenibacillus species* in the cerebrospinal fluid of 44% of infants presenting for neurosurgical evaluation with findings consistent with postinfectious hydrocephalus (PIH) in Eastern Uganda. Here we sought to compare outcomes among hydrocephalic infants with and without *Paenibacillus* detection at the time of hydrocephalus surgery.

**Figure:**
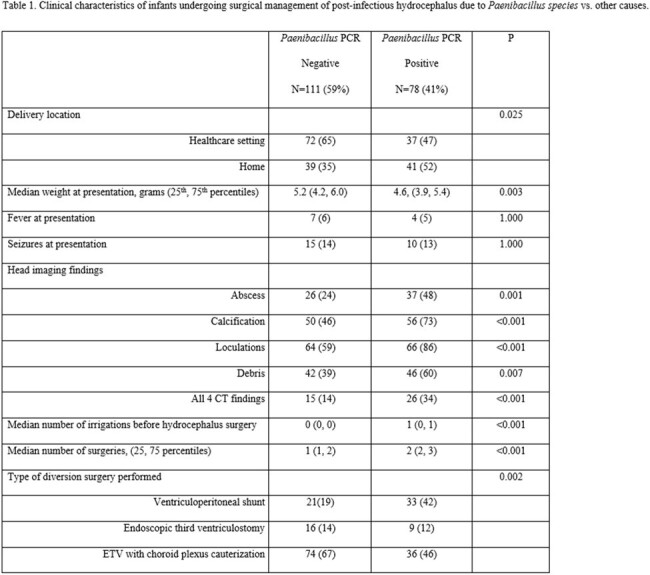

**Methods:**

In a prospective observational trial, 78 infants with PIH who underwent a cerebrospinal fluid (CSF) diversion had a positive CSF polymerase chain reaction result for *Paenibacillus* species (PP), and 111 had a negative result (PN). The primary outcome was diversion failure-free survival defined as being alive without diversion failure at the end of the observation period adjusted for age at the time of diversion and surgery type. Secondary outcomes included overall survival and diversion success.

**Figure:**
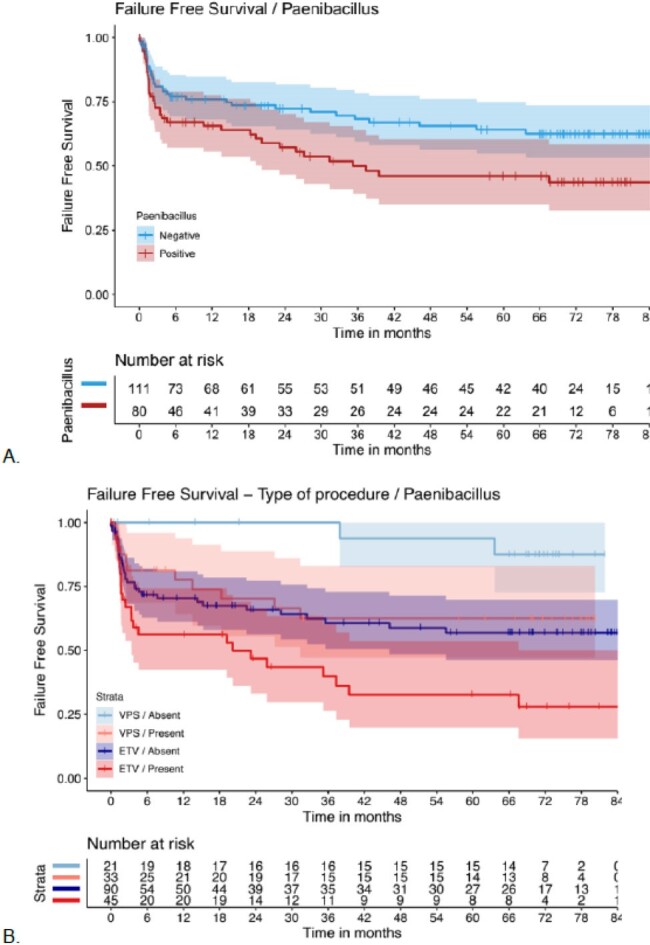

**Results:**

After a median follow-up period of 35.7 months, the primary outcome had occurred in 42 PP patients (54%) and in 76 PN patients (68%) (adjusted hazard ratio (aHR), 2.45; 95% confidence interval [CI], 1.42 to 4.22; P=0.001). PP patients who underwent endoscopic diversion had the worst primary event rate (aHR, 6.47; 95% CI, 2.40 to 17.42; P< 0.001). Death from any cause occurred in 16 PP patients (20%) and 9 PN patients (8%) (aHR, 3.47; 95% CI, 1.44 to 8.37; P=0.006). Diversion failure occurred in 28 PP patients (36%) and in 29 PN patients (26%) (aHR, 2.24; 95% CI, 1.31 to 3.85; P=0.003).

**Conclusion:**

*Paenibacillus* detection in the CSF at the time of hydrocephalus surgery was associated with a significantly increased rate of the composite of diversion failure or death, death, and diversion failure, and was particularly increased for patients who had an endoscopic diversion. Preoperative testing and treatment of *Paenibacillus* infections may improve outcomes for surgical management of PIH.

**Disclosures:**

**Jessica E. Ericson, MD, MPH**, Abbvie: Advisor/Consultant

